# Hub structure in functional network of EEG signals supporting high cognitive functions in older individuals

**DOI:** 10.3389/fnagi.2023.1130428

**Published:** 2023-04-17

**Authors:** Mayuna Tobe, Sou Nobukawa, Kimiko Mizukami, Megumi Kawaguchi, Masato Higashima, Yuji Tanaka, Teruya Yamanishi, Tetsuya Takahashi

**Affiliations:** ^1^Graduate School of Information and Computer Science, Chiba Institute of Technology, Narashino, Japan; ^2^Research Center for Mathematical Engineering, Chiba Institute of Technology, Narashino, Japan; ^3^Department of Preventive Intervention for Psychiatric Disorders, National Institute of Mental Health, National Center of Neurology and Psychiatry, Tokyo, Japan; ^4^Faculty of Medicine, Institute of Medical, Pharmaceutical and Health Sciences, Kanazawa University, Kanazawa, Japan; ^5^Department of Nursing, Faculty of Medical Sciences, University of Fukui, Yoshida, Japan; ^6^Medical Corporation Aokikai Seiwa Hospital, Kanazawa, Japan; ^7^Akashi Mind Hospital, Akashi, Japan; ^8^Faculty of Data Science, Osaka Seikei University, Osaka, Japan; ^9^Research Center for Child Mental Development, Kanazawa University, Kanazawa, Japan; ^10^Department of Neuropsychiatry, Faculty of Medical Sciences, University of Fukui, Yoshida, Japan; ^11^Uozu Shinkei Sanatorium, Uozu, Japan

**Keywords:** aging, betweenness centrality, cognitive function, electroencephalogram, functional connectivity, phase lag index

## Abstract

**Introduction:**

Maintaining high cognitive functions is desirable for “wellbeing” in old age and is particularly relevant to a super-aging society. According to their individual cognitive functions, optimal intervention for older individuals facilitates the maintenance of cognitive functions. Cognitive function is a result of whole-brain interactions. These interactions are reflected in several measures in graph theory analysis for the topological characteristics of functional connectivity. Betweenness centrality (BC), which can identify the “hub” node, i.e., the most important node affecting whole-brain network activity, may be appropriate for capturing whole-brain interactions. During the past decade, BC has been applied to capture changes in brain networks related to cognitive deficits arising from pathological conditions. In this study, we hypothesized that the hub structure of functional networks would reflect cognitive function, even in healthy elderly individuals.

**Method:**

To test this hypothesis, based on the BC value of the functional connectivity obtained using the phase lag index from the electroencephalogram under the eyes closed resting state, we examined the relationship between the BC value and cognitive function measured using the Five Cognitive Functions test total score.

**Results:**

We found a significant positive correlation of BC with cognitive functioning and a significant enhancement in the BC value of individuals with high cognitive functioning, particularly in the frontal theta network.

**Discussion:**

The hub structure may reflect the sophisticated integration and transmission of information in whole-brain networks to support high-level cognitive function. Our findings may contribute to the development of biomarkers for assessing cognitive function, enabling optimal interventions for maintaining cognitive function in older individuals.

## 1. Introduction

In a super-aging society, extending a healthy life expectancy is a pressing issue (Muramatsu and Akiyama, 2011). Physical and mental health are maintained through social participation (Chiao et al., 2013). These activities are supported by high cognitive functioning; however, aging and dementia lead to cognitive decline (Deary et al., 2009), which prevents a physically and mentally healthy lifestyle (Deary et al., 2009). Interventions for dementia can prevent the progression of cognitive decline. Therefore, early detection is important for early intervention (Sanford, 2017; Porsteinsson et al., 2021). Medical interviews and cognitive function tests have been widely used clinically to estimate the cognitive decline in older adults (Boccardi et al., 2016; Arvanitakis et al., 2019). In addition to these methods, the development of biomarkers may allow the evaluation of cognitive functions in a more multifaceted manner (Soria Lopez et al., 2019; Khan et al., 2020). Furthermore, estimating cognitive functions may be utilized not only for intervention for dementia but also for optimal intervention for healthy life for elderly people, according to their individual cognitive functions. Such interventions for enhancing cognitive functions may facilitate achieving “wellbeing” in old age (Bauermeister and Bunce, 2015; Nuzum et al., 2020).

Recently, with the development of methods that can measure brain activity non-invasively, such as functional magnetic resonance imaging, electroencephalography (EEG), and positron-emission tomography, studies to clarify brain functions from brain activity have been conducted widely (Shibasaki, 2008). In particular, EEG has the advantages of low cost, user-friendliness, and high temporal resolution compared to other methods and has wide clinical and healthcare applications (van Diessen et al., 2015). Previous studies have shown that people with age-related diseases exhibit abnormalities in EEG (see review, Jeong, 2004; Yang et al., 2019). For example, wave slowing is observed in Alzheimer's disease (AD), where there is a shift from high-frequency components such as alpha, beta, and gamma to low-frequency components (Cassani et al., 2018). In cases of mild cognitive impairment, patients show an increase in theta power (Prichep et al., 2006; Moretti et al., 2013) as well as a decrease in alpha power (Huang et al., 2000; López et al., 2014). Not only in individuals with pathological conditions but also in healthy individuals, band-specific neural activity on EEG reflects various brain functions (reviewed in Helfrich et al., 2019). The phase component of alpha-band activity supports perceptual functions (De Graaf et al., 2013; Spaak et al., 2014; Helfrich et al., 2017), whereas in higher cognitive functions, typified as attention and prediction, neural activity at the delta and theta bands coordinates the top-down control of cognitive processing (Landau and Fries, 2012; Fiebelkorn et al., 2013; Dugué et al., 2015).

Compared with power spectrum analysis, which examines the activity in intra-brain regions, functional connectivity analysis focuses on the interactions between brain regions (see review, Fingelkurts et al., 2005). Functional connectivity, which is defined as the degree of synchronization and information flow between neurally activated areas of different brain regions (strong synchronization and large information flow corresponding to strong functional connectivity), has been widely utilized for evaluating pathological and aging brain network changes and the brain network characteristics that support cognitive functions in healthy individuals (Stam et al., 2007a; Zhou and Seeley, 2014; Sala-Llonch et al., 2015; Torres-Simón et al., 2022). In particular, the relationship between functional connectivity and cognitive status has been elucidated in diverse dementia cases involving mild cognitive impairment, AD, and dementia with Lewy bodies (Zhou and Seeley, 2014; van Dellen et al., 2015; Zhang et al., 2016; Nobukawa et al., 2020a; Torres-Simón et al., 2022). In this relationship, most of the reduced functional connectivity correlates with decreasing cognitive functions (Zhou and Seeley, 2014; van Dellen et al., 2015; Nobukawa et al., 2020a). However, some functional connectivity is enhanced, especially in mild cognitive impairments (Zhang et al., 2016). Therefore, to estimate the cognitive state in older individuals, capturing disease-specific (Zhou and Seeley, 2014; van Dellen et al., 2015; Zhang et al., 2016; Nobukawa et al., 2020a; Torres-Simón et al., 2022), age-specific (Scally et al., 2018; Ando et al., 2022), and level-of-cognitive-functional-specific (Nobukawa et al., 2020b) spatial patterns of functional connectivity is important. Moreover, among the many approaches to estimate functional connectivity, such as coherence measure and transform entropy, instantaneous phase synchronization, typified as the phase lag index (PLI), is an effective approach for estimating functional connectivity with higher spatial resolution compared to other synchronization approaches, by virtue of suppressing the influence of volume conduction (Stam et al., 2007b; Tobe and Nobukawa, 2022). In particular, utilizing this approach, older people have been reported to experience reduced connectivity in the upper alpha band compared to younger adults, even in EEG signals that have relatively low spatial resolution (Scally et al., 2018). Additionally, AD patients have shown a decrease in functional connectivity in the alpha and beta bands (Stam et al., 2009; Engels et al., 2015; Nobukawa et al., 2020a), whereas healthy older individuals with high cognitive functions exhibit strong whole-brain functional connectivity at the alpha band (Nobukawa et al., 2020b).

Functional connectivity examines the pair-wise interactions of neural activity. By contrast, graph theory analysis, which can examine global topological features consisting of an assembly of functional connectivities, has been widely applied to explore whole-brain network abnormalities in many diseases (Rubinov and Sporns, 2010; Sporns, 2018). For example, in AD, alterations in topological network characteristics, such as the loss of small-world characteristics, have been reported (Stam et al., 2007b; de Haan et al., 2009). In addition, these alterations, typified by the clustering coefficient and nodal centrality, appear even in healthy aging individuals (Knyazev et al., 2015; Javaid et al., 2022). Of the different kinds of topological features, the brain region termed a “hub,” i.e., the most important brain region affecting the activity of the whole network, is an essential factor coordinating whole-brain interactions. In particular, betweenness centrality (BC), which is defined as centrality based on the shortest paths in the network, is widely used to detect the “hub” in a functional network (Liu et al., 2014; van Oort et al., 2014; Engels et al., 2015). By virtue of focusing on pathways among whole brain regions, BC can easily capture global “hub” structures (Liu et al., 2014; van Oort et al., 2014; Engels et al., 2015). Cognitive function emerges by whole-brain interactions and their integration among many brain regions (van den Heuvel and Sporns, 2013). Thus, BC is an effective candidate to reveal the global topological features related to cognitive functions.

Evaluation of the hub structure in functional networks related to aging and age-related pathology has been proceeding (Engels et al., 2015). In particular, Engels et al. (2015) showed that the alternation of BC was related to the stage of AD severity and demonstrated the effectiveness of usage of BC in the evaluation of pathological cognitive deficits. Knyazev et al. (2015) and Javaid et al. (2022) demonstrated that under healthy aging conditions the hub structure in the functional networks decreases with aging. However, Knyazev et al. (2015) captured the hub structure by modularity based on the average among adjacent connectivity [called as node degree (ND)], instead of pathways among whole brain regions as in BC. In the study by Javaid et al. (2022), frequency-band-specific functional network was not identified, although BC was evaluated. Moreover, in these previous studies (Knyazev et al., 2015; Javaid et al., 2022), the relationship between alteration in cognitive functions with aging and hub structure remains unclear.

In this context, we hypothesized that the global hub structure of functional networks reflects cognitive function in healthy elderly individuals. To investigate this hypothesis, we examined the relationship between the hub structure obtained by BC values in functional networks estimated from EEG using PLI and cognitive functions in healthy older individuals, measured using the Five Cognitive Functions test (Five-Cog test) total score (Miyamoto et al., 2009). From the view point of assessment of cognitive function for social implementations, easier measurement method is required. Moreover, previous studies reported that even in the resting state, performance of cognitive functions reflect the functional connectivity and its topology (Engels et al., 2015; Nobukawa et al., 2020b). Therefore, we evaluated the EEG signals under the eyes closed resting state.

## 2. Materials and methods

### 2.1. Participants

In this study, participants were recruited from among older adults living in the community in Eiheiji-Cho, Japan. We selected 38 medication-free, healthy, older participants for this study. Table 1 shows the descriptive statistics and demographic information of the participants. The sample size for this study was determined based on those used in previous studies on the relationship between the evaluation index at the electrode level and cognitive functions (Nobukawa et al., 2020b; Ando et al., 2022; Iinuma et al., 2022). We excluded individuals with major medical or neurological conditions, a history of alcohol or drug dependency, and systemic diseases, including hypertension, hyperlipidemia, and diabetes mellitus. To quantify the degree of cognitive function in each individual, these participants underwent cognitive function tests, such as the Five-Cog test (Miyamoto et al., 2009) and the mini mental state examination test (MMSE) (Folstein et al., 1975). None of the participants had an MMSE test score lower than the dementia threshold of 24, indicating that there were no patients with dementia in this study. In this study, the results of the Five-Cog test were used as a quantification index for the degree of cognitive function and the participants were divided into two groups, high- and low-cognitive function groups, according to the score. The Five-Cog test was developed as a cognitive function test for mass examination by video among older people in Japan (Fujii et al., 2021). The stimuli and instructions for the test were projected on a screen and the examinee followed the images and filled in the response form in pencil. The Five-Cog test is composed of six items, including five categories of cognitive tasks (i.e., attention, memory, visuospatial function, language, and reasoning) and a finger movement task (Miyamoto et al., 2009; Kamegaya et al., 2012, 2014; Sugiyama et al., 2015; Fujii et al., 2021). The sum of the scores for each category was used as a measure of cognitive function (the detailed explanation for each task and its sub-score is shown in Supplementary material). The Five-Cog test is similar to MMSE, because it is used as a screening test for dementia along with MMSE. However, the Five-Cog test in comparison with MMSE can assess a more extensive range of cognitive levels between dementia to healthy cognitive level by virtue of avoiding ceiling effect (Miyamoto et al., 2009; Kamegaya et al., 2012, 2014; Fujii et al., 2021). Based on the median (101.5 points) for the distribution of scores, the participants were then divided into two groups: participants with 101 points or less were allocated to the low-cognitive function group, and those with 102 points or more were allocated to the high-cognitive function group. The high-cognitive function group consisted of 6 men and 13 women (average age, 69.05 years; standard deviation [SD]), 3.03 years; range, 65–74 years). The low-cognitive function group consisted of 6 men and 13 women (average age, 72.63 years; SD, 3.25 years; range, 67–78 years). Characteristics of the participants in the two groups are presented in Table 2. The age and education history were significantly different between these groups. All participants provided informed consent before the start of the study. The study protocol was in agreement with the Declaration of Helsinki and approved by the Ethics Committee of the University of Fukui. The data used in this study were evaluated in our previous study for complexity analysis of EEG. However, the age distribution in this study was more restricted than that in our previous study, to ensure a more rigid evaluation (Iinuma et al., 2022).

**Table 1:** Descriptive statistics and demographic information of the total participants.

**Variables**	
Mean age [Standard deviation (SD)], years	70.84 (3.59)
Mean education history [Standard deviation (SD)], years	12.08 (2.01)
Mean total score of Five Cognitive Functions test (Five-Cog) [Standard deviation (SD)]	101.11 (15.31)
Mean Mini Mental State Examination (MMSE) [Standard deviation (SD)]	28.76 (1.13)
Mean body mass index values (kg/m^2)	23.48
Mean blood pressure (Systolic, mmHg)	137.63
Mean blood pressure (Diastolic, mmHg)	80.03
Male/Female	12/26

**Table 2:** Descriptive statistics and demographic information.

	**High-cognitive function group**	**Low-cognitive function group**	***P*-value**
Mean age [Standard deviation (SD)], years	**69.05 (3.03)**	**72.63 (3.25)**	**0.0012**
Mean education history [Standard deviation (SD)], years	**12.74 (1.52)**	**11.42 (2.24)**	**0.0414**
Mean total score of Five Cognitive Functions test (Five-Cog) [Standard deviation (SD)]	**113.26 (9.27)**	**88.95 (9.13)**	<**0.001**
Mean Mini Mental State Examination (MMSE) [Standard deviation (SD)]	28.95 (0.97)	28.58 (1.26)	0.32
Mean body mass index values (kg/m^2)	23.75	23.21	0.64
Mean blood pressure (Systolic, mmHg)	137.47	137.79	0.95
Mean blood pressure (Diastolic, mmHg)	80	80.05	0.99
Male/Female	6/13	6/13	1.0

For clarity, values with *p* < 0.05 are shown in bold.

### 2.2. EEG recording

EEG signals were recorded using a 21-channel electroencephalography system (EEG-4518; Nihon-Koden, Tokyo, Japan). With the electrode arrangement based on the International 10–20 system, EEG was recorded from 19 electrodes (Fp1, Fp2, F3, F4, C3, C4, P3, P4, O1, O2, F7, F8, T3, T4, T5, T6, Fz, Cz, and Pz) equally distributed across the scalp, using the two ear lobes jointly as the reference. Based on previous studies, even with this low-density EEG signal, it was reported that functional connectivity regarding cognitive functions could be elucidated (Nobukawa et al., 2020b). All the participants were studied while seated in a soundproof, electrically shielded, light-controlled recording room. During the EEG recording, they were in a state of wakefulness, with their eyes closed and rested for at least 3 min. The sampling frequency of recordings was 500 Hz. The time constant was 0.3 s. A bandpass filter was applied from 1 to 60 Hz. Because the line noise located at 60 Hz is the upper limit of the bandpass filter, a notch filter was not applied. The electrooculogram and electromyograms were recorded along with EEG measurements. The electrode impedance was controlled to less than 10 kΩ for each electrode using the recording device and its software with a self-check function. Artifacts caused by several factors, such as eye movements, blinks, and muscle activity, were manually excluded from the evaluated epochs by focusing on specific artifact patterns and monitoring the electrooculogram and electromyograms. In this study, we removed durations with artifacts at even one channel from the analyzed epochs. Therefore, in all epochs used in this analysis, EEG signals from 19 channels were obtained.

### 2.3. Phase lag index

PLI evaluates the functional connectivity between two time series (Stam et al., 2007b). PLI is based on the asymmetry of the phase-difference distribution between the two time series, which is determined using the Hilbert transform. Designed to ignore the zero and π phase differences, the PLI can reduce the influence of volume conduction. The PLI can be obtained from a time series of phase differences Δφ with *t*_*k*_, as follows:


(1) $$\begin{array}{l} PLI=|<\text{sign}\left(\Delta \varphi \left(t_{k}\right)\right)>|, \end{array}$$

where “sign” represents a signum function, < > indicates the mean values, and || denotes the absolute values. *t*_*k*_ represents the time series (*k* = 1, 2 …, *N*). PLI values ranged from 0 to 1. A value of 0 means no coupling or coupling with zero lag, while a value of 1 means perfect phase coupling.

In PLI analyses, the values decrease with increasing epoch length (Fraschini et al., 2016); therefore, it is difficult to identify changes with increasing epoch length. Moreover, to increase the size of the obtained artifact-free epochs, the epoch length must be shortened. In addition, using short epoch lengths makes it impossible to capture information on slow-frequency components. To balance these considerations, we used an epoch length of 4 s. The PLI values for each participant were averaged over epochs. In addition, as the number of epochs differed for each individual, the PLI value for each epoch was obtained and averaged for each participant (mean number of epochs among individuals: 75.03, maximum number of epochs among individuals: 135, minimum number of epochs among individuals: 22). The statistical values in high- and low-cognitives are shown in Table 3. No significantly large difference between high- and low-cognitive function groups (*t* = 0.978, *p* = 0.334) was confirmed. (Positive *t*-value corresponds to larger epoch size of high-cognitive function groups rather than one for low-cognitive function group). These epoch sizes were satisfactory for stabilizing the PLI value through averaging the epochs (Nobukawa et al., 2020a; Ando et al., 2022).

**Table 3:** Mean value, median value, and standard deviation of epoch size in high- and low-cognitive function groups.

	**Mean value**	**Median value**	**Standard deviation**
High-cognitive function group	79.63	86	26.47
Low-cognitive function group	70.42	68	31.38

No significant large difference between high- and low-cognitive function groups (*t* = 0.978, *p* = 0.334) was confirmed. (Positive *t*-value corresponds to larger epoch size of high-cognitive function groups than one for low-cognitive function group).

With regards to the flow for calculating the PLI values, first, the time-series signal in each electrode was divided into four bands by finite impulse response (FIR) filter with linear phase: delta (2–4 Hz), theta (4–8 Hz), alpha (8–13 Hz), and beta (13–30 Hz). Here, the roll-off of filtering process of the recording system, EEG-4518 appear in ≳57 Hz; therefore, we did not use the gamma band. Subsequently, a square 19 × 19-weighted adjacency matrix for the PLI value was constructed by computing the PLI values between all pair-wise combinations of 19 electrodes for each epoch in each frequency band. The PLI was calculated using the HERMES toolbox (Niso et al., 2013).

### 2.4. Betweenness centrality

BC is widely used to identify focal nodes in brain networks (Freeman, 1978). Mathematically, the BC is defined as the fraction of the number of shortest paths that pass through a given node to the total number of shortest paths in the network. A node with a high BC value corresponds to a bridge node in the network and plays an important role as a hub. The BC value *b*_*i*_ of node *i* is defined as:


(2) $$b_{i}=\frac{1}{\left(n-1)(n-2\right)}\sum_{h,j\in N,h\neq i,j\neq i}\frac{\rho_{hj}(i)}{\rho_{hj}},$$

where ρ_*hj*_ is the number of shortest paths from node *h* to *j*, and ρ_*hj*_(*i*) is the number of paths passing through node *i*. *N* is the set of all nodes in the network, and *n* is the number of nodes. Subsequently, *b*_*i*_ was normalized between 0 and 1 by dividing it by (*n*−1)(*n*−2). In BC estimation of functional connectivity, the length of the pathway between nodes is defined as an inverse number of PLI values. Moreover, to allow focusing on the main backbone of the network pathway, long pathways were pruned by using the minimum spanning-tree process; the study adopted this pruning process (Engels et al., 2015; van Dellen et al., 2018). This network was evaluated for BC analysis instead of a fully connected network. To calculate BC, we applied the MATLAB Brain Connectivity Toolbox (Rubinov and Sporns, 2010).

### 2.5. Statistical analysis

Spearman's correlation analysis was performed to analyze the relationships between Five-Cog total scores and BC values, between age and BC values, and between education history and BC values. Against BC values exhibiting significantly strong correlation with Five-Cog total scores, multiple linear regression analysis with Five-Cog total score, age, education history, and sex as explanatory variables was conducted. In these analysis, the statistical significance level was set at *p* < 0.05.

For the statistical comparison of BC values between the low- and high-cognitive function groups, a repeated-measures analysis of covariance (ANCOVA) was performed at each frequency band. The within-participants factor was 19 electrodes, the between-participants factor was group, and the covariates were age and educational history. Correction of the degrees of freedom was made by Greenhouse–Geisser adjustment and results were considered significant at a two-tailed α level of 0.05. *Post-hoc* *t*-tests were conducted to assess the significant main effects of group and electrode-wise interactions. To control for multiple comparisons, false discovery rate (FDR) correction was applied with a threshold of *q* = 0.05 using the Benjamini–Hochberg method to *t*-scores (Benjamini and Hochberg, 1995).

Statistical analyses were performed using MATLAB version R2022b (Natick, MA, USA) and SPSS software version 28.0 (IBM SPSS Inc., Armonk, NY, USA).

## 3. Results

### 3.1. Betweenness centrality: Multiple linear regression analysis

**Figure 1:**
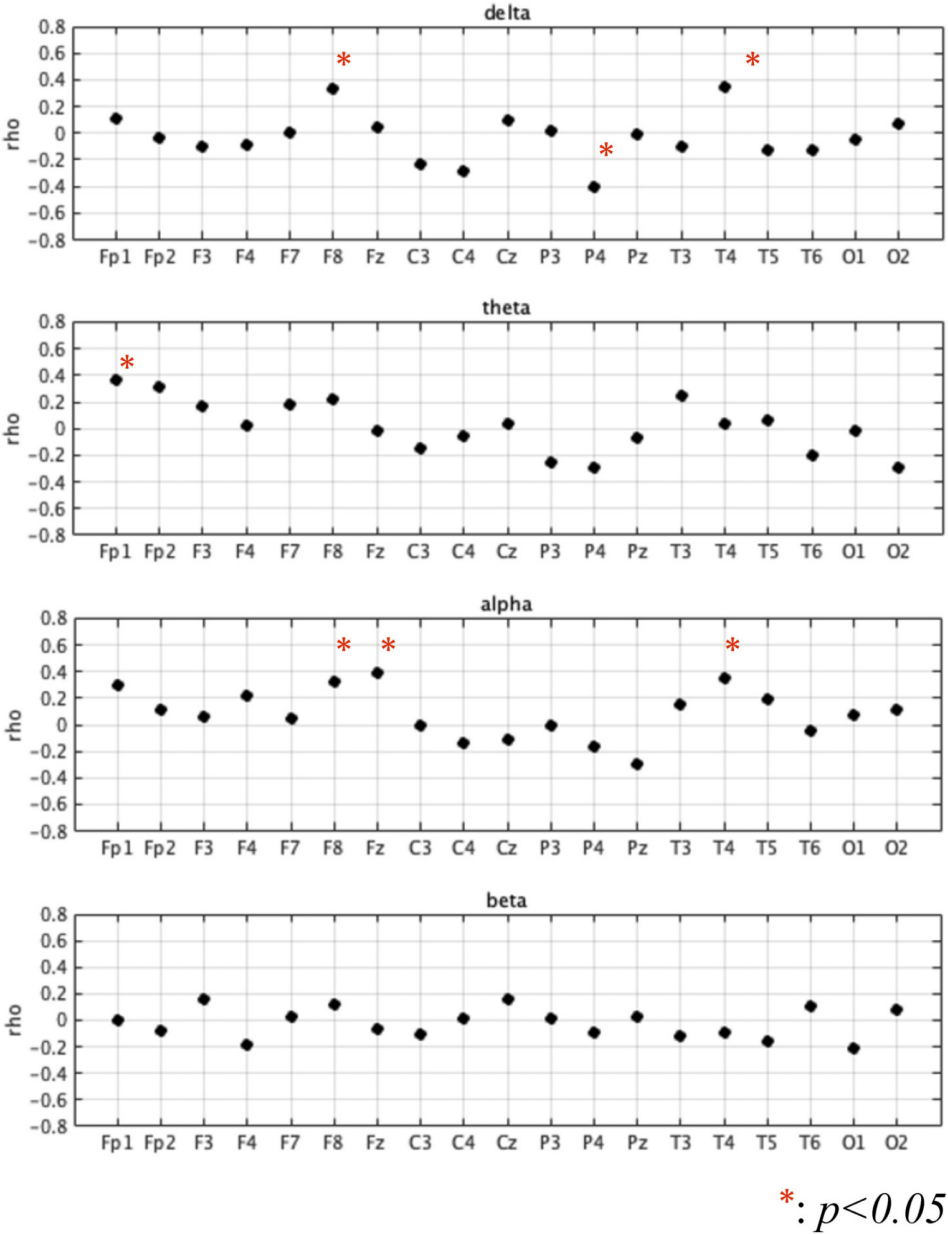
Correlation coefficient (rho) between betweenness centrality (BC) values and Five Cognitive Functions test (Five-Cog) total score for each of 4 frequency bands (delta (2–4 Hz), theta (4–8 Hz), alpha (8–13 Hz), and beta (13–30 Hz)) and 19 electrodes. Statistically significant strong positive correlations were observed at F8 [rho = 0.335 (*p* = 0.039)] and T4 [rho = 0.343 (*p* = 0.034)] in the delta band, Fp1 [rho = 0.368 (*p* = 0.022)] in the theta band, and F8 [rho = 0.325 (*p* = 0.046)] and Fz [rho = 0.394 (*p* = 0.014)] in the alpha band. In contrast, statistically significant strong negative correlation of the Five-Cog total score with P4 [rho = −0.40 (*p* = 0.012)] was observed in the delta band.

**Figure 2:**
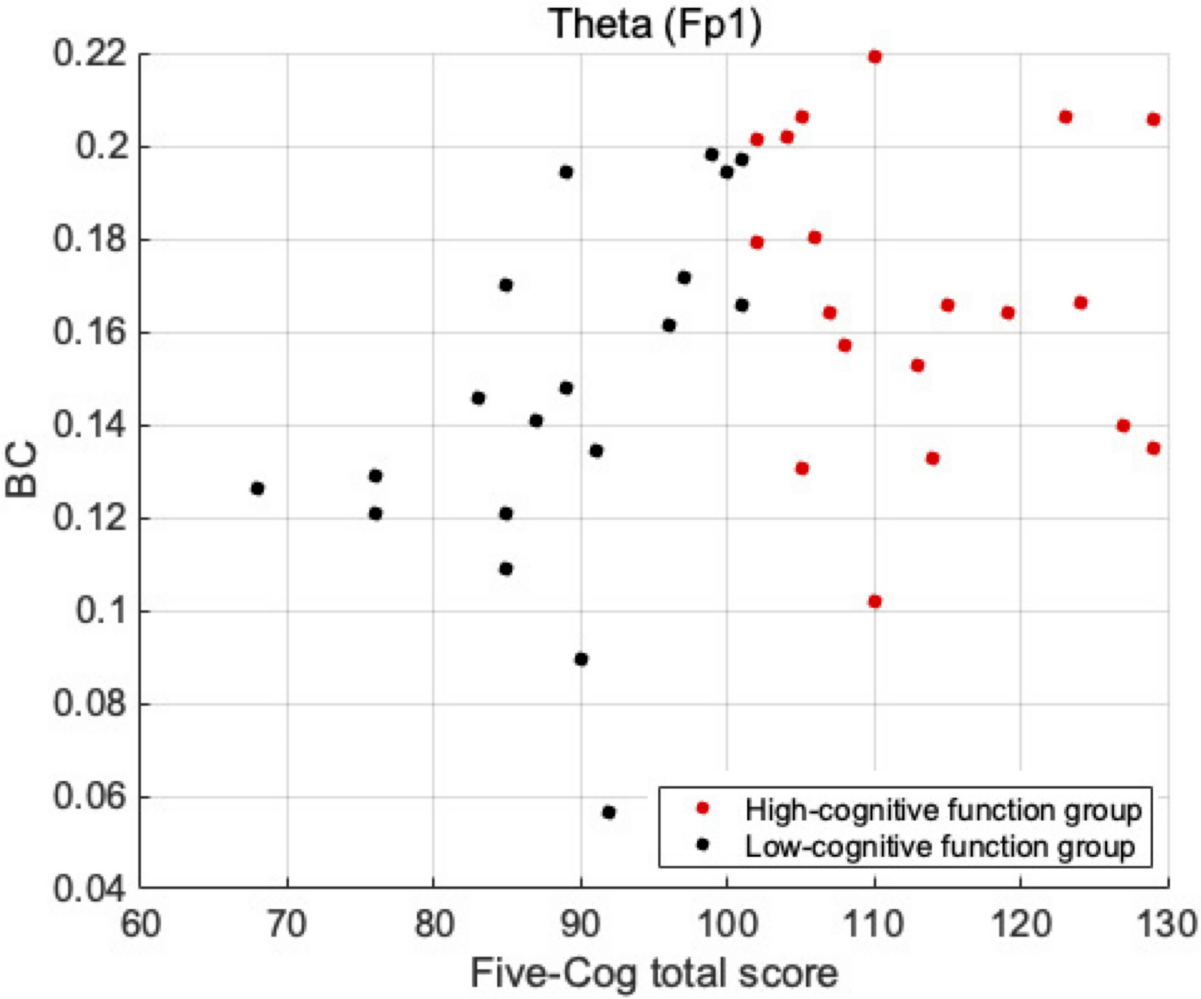
Scatter plot between BC values and Five-Cog total scores at Fp1 in the theta band, which exhibited significant large β for Five-Cog total score in Table 4. The result showed the different tendency of BC distribution between high- and low-cognitive function groups.

First, we investigated demographic characteristics, BC, Five-Cog total score, age, and education history. Figure 1 shows the correlation coefficient between the BC values and Five-Cog total score for 4 frequency bands and 19 electrodes. Statistically significant strong positive correlations were observed at F8 [rho = 0.335 (*p* = 0.039)] and T4 [rho = 0.343 (*p* = 0.034)] in the delta band, Fp1 [rho = 0.368 (*p* = 0.022)] in the theta band, and F8 [rho = 0.325 (*p* = 0.046)] and Fz [rho = 0.394 (*p* = 0.014)] in the alpha band. In contrast, statistically significant strong negative correlation of the Five-Cog total score with P4 [rho = −0.40 (*p* = 0.012)] was observed in the delta band. Statistically significant strong positive and negative correlations appeared at several electrodes in the delta and theta bands between the BC values and age and between the BC values and education history (data not shown). Against the BC values that exhibited significant strong correlation with Five-Cog total score, multiple linear regression analysis with Five-Cog total score, age, education history, and sex was conducted as shown in Table 4. Significant large multiple correlation coefficients *R*, *R*^2^, and *F* were observed at F8 and T4 in the delta band and Fp1 in the theta band. Among these BC values, the BC values at T4 in the delta band and at Fp1 in the theta band exhibited significantly strong negative β values for age and positive β values for Five-Cog total score, respectively. The scatter plot with Five-Cog total score at Fp1 in the theta band is shown in Figure 2.

**Table 4:** Summary of multiple linear regression analysis for betweenness centrality (BC).

	***R***	***R* ^2^**	***F*-vale (*p*-value)**	**β for Five-Cog total score (*p*-value)**	**β for age (*p*-value)**	**β for education history (*p*-value)**	**β for sex (*p*-value)**
**Delta**							
F8	**0.51**	**0.263**	**2.947 (0.035)**	0.260 (0.160)	−0.231 (0.188)	0.139 (0.425)	0.075 (0.637)
P4	0.447	0.200	2.061 (0.109)	–	–	–	–
T4	**0.549**	**0.301**	**3.554 (0.016)**	0.285 (0.115)	−**3.67 (0.035)**	−0.326 (0.060)	−0.092 (0.552)
**Theta**							
Fp1	**0.496**	**0.246**	**2.692 (0.048)**	**0.538 (0.006)**	0.136 (0.440)	−0.225 (0.203)	−0.184 (0.258)
**Alpha**							
F8	0.446	0.199	2.050 (0.110)	–	–	–	–
Fz	0.426	0.182	1.834 (0.146)	–	–	–	–
T4	0.344	0.119	1.110 (0.368)	–	–	–	–

For clarity, values with *p* < 0.05 are shown in bold. *R*, multiple correlation coefficient; *R*^2^, coefficient of determination; β, standardized partial regression coefficient.

### 3.2. Betweenness centrality: Group comparisons between the high- and low-cognitive function groups

**Figure 3:**
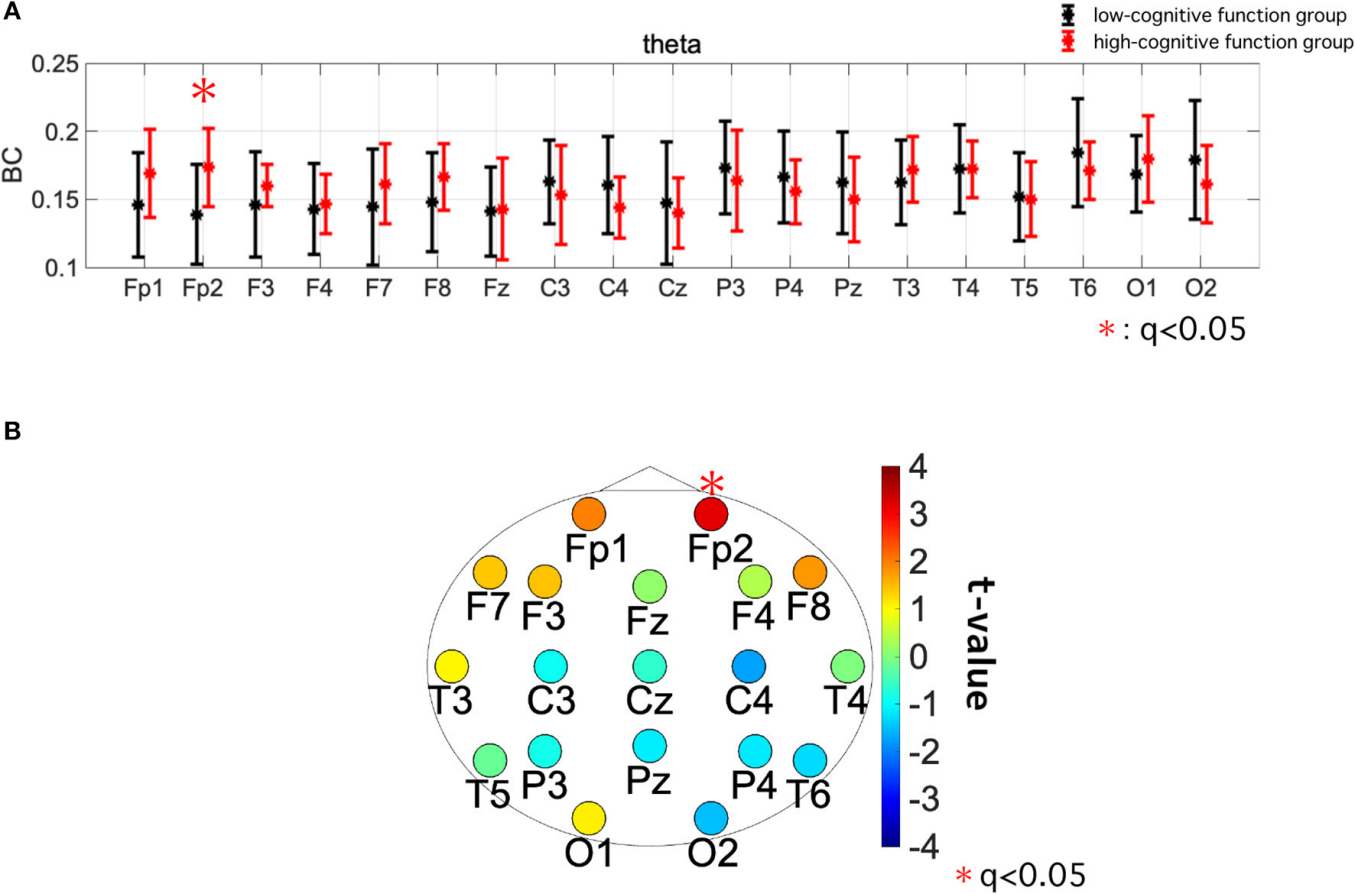
**(A)** Mean value and standard deviation of betweenness centrality (BC) within each group (high- and low-cognitive function groups) in the theta band. The dots and error bars represent the mean and standard deviation, respectively. **(B)** The topography of *t*-values of the value of BC in the theta band. Warm colors indicate that the high-cognitive function group has a greater value than the low-cognitive function group. The significantly greater BC value at Fp2 for high-cognitive function than low-cognitive function [*t* = 3.23 (*p* = 0.0026)], which satisfied with criteria of FDR (*q* < 0.05), was confirmed.

Table 5 summarizes the results of the repeated-measures ANCOVA for BC, with age and educational history as covariates. We identified both a significant group effect and group × electrode interaction effect in the theta band. Figures 3A, B show the mean value of BC in each group for the theta band and the results of the *post-hoc* *t*-test, respectively. The significantly greater BC value at Fp2 for high-cognitive function than low-cognitive function [*t* = 3.23 (*p* = 0.0026)], which satisfied the criteria of FDR (*q* < 0.05), was confirmed.

**Table 5:** The results of the repeated-measure analysis of covariance between the high- and low-cognitive function groups for the value of betweenness centrality, using age and educational history as covariates.

	**Group effect**	**Group × electrode**
Delta	F = 0.007 (p = 0.932,η^2^ = 0.000)	F = 0.736 (p = 0.704, / η = 0.021)/
Theta	F = 5.148 (p = 0.030,η ^2^ = 0.131)	F = 2.726 (p = 0.004,η ^2^ = 0.074)
Alpha	F = 0.951 (p = 0.336, /η^2^ = 0.027)/	F = 1.364 (p = 0.226, /η^2^ = 0.039)/
Beta	F = 1.268 (p = 0.268, /η^2^ = 0.036)/	F = 1.000 (p = 0.439, /η^2^ = 0.029)/

F, p, and η^2^ value with *p* < 0.05 are represented in bold text.

## 4. Discussion

In this study, we investigated the relationship between cognitive function level and hub properties in a functional network structure in healthy older participants using the BC of PLI in EEG signals. Through multiple linear regression analysis, statistically significant positive correlations of Five-Cog total scores were found mainly in the frontal regions and in the theta band. Additionally, a comparison of BC between the high- and low-cognitive function groups revealed significant group differences with electrode dependence in the theta band. In particular, a high BC was observed in the high-cognitive function group in the frontal region.

First, we considered the region-specificity of changes in BC in terms of cognitive functions, particularly in the frontal region in the theta band. Regarding the frequency bands, many previous studies have revealed that the theta band is related to cognitive performance (Klimesch, 1999). In particular, many studies have been conducted on the relationship between theta activity and cognitive functions, and it has been shown that theta power is related to cognitive functions such as working memory control (Sauseng et al., 2005; Hsieh and Ranganath, 2014), cognitive control (Nigbur et al., 2011; Zavala et al., 2018), and attention (Aftanas and Golocheikine, 2001). Global neural interactions, which exhibit functional connectivity in the theta band, more strongly reflect the degree of cognitive function than local regional activity represented by the intra-regional power component (Lejko et al., 2020; Nobukawa et al., 2020b). Functional networks in the frontal regions of the theta band are involved in top-down control of cognitive processing (Von Stein and Sarnthein, 2000; Min and Park, 2010). This sophisticated top-down control approach supports high cognitive functioning (Edin et al., 2009; Jo et al., 2019). Moreover, recent studies showed that the top-down projections of frontal-polar structural/functional connectivity support the extensive cognitive functions (Souza et al., 2022) (reviewed in Tsujimoto et al., 2011). The high frontal BC in the theta band obtained in this study (see Figures 2, 3 and Tables 4, 5) implies the existence of this top-down control, which achieves high cognitive functioning. Regarding the lack of correlation with cognitive functions and group difference in the faster bands, the neural activities at fast frequency bands do not form long-range functional connections of extensive cognitive functions in comparison with slow frequency bands (reviewed in Ishii et al., 2017). Additionally, in extensive cognitive functions involving attention and prediction, which are measured by the Five-Cog test, the neural activity at the delta and theta bands, rather than the faster bands, coordinate the top-down control of cognitive processing, (Landau and Fries, 2012; Fiebelkorn et al., 2013; Dugué et al., 2015). Therefore, in the faster bands the relationship of BC with cognitive functions did not appear.

Second, we considered the effectiveness of BC in analyzing functional networks related to cognitive function. Because BC quantifies the ratio of the shortest path between node pairs that pass through the node of interest (Freeman, 1978), it can be used to evaluate the global propagation of neural signals generated by whole-brain interactions in comparison with pair-wise functional connectivity for directly adjacent brain regions (see the results of pair-wise PLI analysis in Supplementary material). BC can be considered a useful approach to capture these global interactions in theta-band networks. In particular, during the last decade, BC has been applied to capture alterations in brain networks for cognitive functions under pathological conditions (Dai and He, 2014; Engels et al., 2015; Yun and Kim, 2021). However, this study showed that BC is useful for capturing the global network topology involving cognitive functions not only under pathological conditions (Dai and He, 2014; Engels et al., 2015; Yun and Kim, 2021) but also in healthy older people. Thus, this study may have opened a new avenue for studies involving the assessment of functional connectivity for healthy cognitive functions.

Third, we must consider the reason why BC captures the hub structure related to the cognitive function, in contrast to ND as the hub structure focused on neighbor electrodes (see the results in the case using ND for Supplementary material). Previous studies by Sporns et al. (2007) and Mišić et al. (2011) examined the relationship between the complexity produced by the interaction of neural activity and the hub structure captured by ND and BC; consequently, a strong correlation was observed between the complexity and hub structure in both cases by ND and BC. In contrast, this study focuses on the relationship between extensive cognitive functions emerging from global interactions (Sporns and Betzel, 2016; Battiston et al., 2020) and hub structures rather than the complexity of neural activity itself involving local and global interactions. Therefore, ND cannot capture the hub structure to produce global interactions because it focuses only on the connectivity between adjacent electrodes (see Supplementary material). Since BC can assess the global hub structure, it enables capturing the relationship with the extensive cognitive functions by global neural interaction. In comparison with the previous studies on healthy aging, this study demonstrated the effectiveness of BC in the evaluation of the global frequency-band specific topological characteristics for the first time; while the previous studies used the indices defined by the adjacent connectivity such as clustering coefficient and nodal centrality (Knyazev et al., 2015; Javaid et al., 2022).

Fourth, we must discuss the comparison with our previous study with the same data set (Iinuma et al., 2022). Based on the findings for complexity analysis of neural activity, global neural interaction increases the slow temporal-scale complexity (Wang et al., 2018). Our previous study showed that the slow temporal-scale complexity increases in the frontal, parietal, and temporal regions (Iinuma et al., 2022). Considering that in the frontal region, the global hub structure related to the top-down cognitive process exists (van den Heuvel and Sporns, 2013; Powers et al., 2016), we speculate that the enhanced complexity at the frontal region emerges by the global neural interactions in the hub structure observed in the theta band.

This study had some limitations. First, regarding the slower functional connectivity analysis, i.e., about the delta band, a previous study reported the alternation of BC in the delta band under the condition of cognitive deficits (Engels et al., 2015); the delta-band frequency network also plays an important role for top-down cognitive processing, in addition to the theta-band network (Landau and Fries, 2012; Fiebelkorn et al., 2013; Dugué et al., 2015). Therefore, more detailed analysis in the delta-band network is important. In this study, the epoch length for calculating the PLI was set to 4 s, but this epoch length may be too short to evaluate delta activity (2–4 Hz) precisely. To reveal the topology of the delta network by PLI, sequential and prolonged artifact-free EEG acquisition may be needed. Second, the index used in this study was unable to detect the direction of neural signal propagation. To reveal the information regarding integration and transmission at the hub of a functional network, it is necessary to evaluate the directional functional connectivity with directed PLI (Stam and van Straaten, 2012). Moreover, many methods for functional connectivity have been proposed, which can remove the influence of volume conduction (Nolte et al., 2004; Pascual-Marqui et al., 2011; Brookes et al., 2012). Therefore, finding the optimal evaluation index to evaluate functional connectivity must be considered. Third, the sample size in this study was considerably small to assess the respective cognitive functions; therefore, a larger sample size is required. Fourth, in terms of social implementation, assessing cognitive function through low-density EEG is important. However, high-density EEG is also essential for a more detailed evaluation of network topology involving other types of topology, such as clustering coefficient and efficiency. Finally, regarding technical issues, Laplace re-reference was appropriate to weaken the common source problem. On the other hand, PLI analysis achieves relatively high spatial resolution without re-reference (Nobukawa et al., 2020b). It has also been pointed out that re-reference is not always necessary, especially in an extended version of PLI called wPLI (Cohen, 2015). However, the actual effect of re-reference in detecting the hub structure at the global topology level has not yet been evaluated; therefore, a detailed verification of the comparison will be necessary in the future.

## 5. Conclusion

In this study, by evaluating functional networks in EEG signals, we identified the hub structure of theta networks that support a wide range of cognitive functions in healthy older people. This structure may reflect the sophisticated integration and transmission of information in whole-brain networks. Furthermore, we demonstrated that BC based on PLI of EEG signals is an effective approach for identifying this topology. Although several limitations remain, our findings may contribute to the development of biomarkers for assessing cognitive function, which is desirable for maintaining high cognitive functioning by optimal intervention for older individuals in the upcoming super-aging society.

## Data availability statement

The original contributions presented in the study are included in the article/Supplementary material, further inquiries can be directed to the corresponding author.

## Ethics statement

The studies involving human participants were reviewed and approved by Ethics Committee of the University of Fukui. The patients/participants provided their written informed consent to participate in this study.

## Author contributions

MT, SN, and TT conceived the methods and wrote the main manuscript and prepared all figures. MT, SN, KM, TY, and TT analyzed and discussed the results. KM, MK, MH, YT, and TT conducted the experiments. All authors contributed to the manuscript revision and have read and approved the submitted version.
