# Supplementary material for: Hub structure in functional network of EEG signals supporting high cognitive functions in older individuals
Source: Front Aging Neurosci. 2023 Apr 17;15:1130428. doi: 10.3389/fnagi.2023.1130428 (PMC10149684; doi:10.3389/fnagi.2023.1130428)
Supplement: Supplementary file 1 [file Data_Sheet_1.PDF]

1 DETAILED SUB-SCORES OF FIVE COGNITIVE FUNCTIONS TEST

The five cognitive functions test (Five-Cog test) was developed as a cognitive function test to use for mass examination by video among older people in Japan (Fujii et al., 2021). The stimuli and instructions for the test were projected on a screen and the examinee followed the images and filled in the response form in pencil. Five-Cog test is composed of six items, including five categories of cognitive tasks (i.e., attention, memory, visuospatial function, language, and reasoning) and a finger movement task. The detailed explanations are as follows:

- As for attention ability, the participants were asked to mark the letters “upper,” “middle,” and “lower” satisfying with correct positions and numbered these letters (letter-position matching task).
- As for memory ability, the participants were asked to memorize 32 words along with a cue category and write down the remembered words corresponding to the presented category (category cued recall task).
- As for visuospatial cognition ability, the participants were asked to draw a clock face and hands on it to indicate 11:10 (clock drawing task).
- As for language ability, the participants were asked to write as many animal names as possible in 2 minutes (word fluency task).
- As for reasoning ability, specifically focusing on analogical thinking, the participants were asked to solve the 16 questions about extracting superordinate concept against the given words within 3 minutes (similarities task).
- As for movement ability, participants were asked to encircle a number as fast as possible in 15 seconds (finger movement task).

In this study, we used the total scores of the Five-Cog test to assess extensive cognitive function. Table S1 shows mean sub-scores of Five-Cog test of total participants [Standard deviation (SD). In dividing two groups, in Table S2, the mean sub-scores of the Five-Cog test in high- and low-cognitive function groups [Standard deviation (SD)] are represented. Compared to the low-cognitive function group, all sub-scores of the high-cognitive function group, except for the clock drawing task, were significantly higher.

Table S1. Mean sub-scores of the Five Cognitive Functions test (Five-Cog) of total participants [Standard deviation (SD)].

Sub-score	
Letter-position matching task	25.18 (5.37)
Category cued recall task	16.26 (3.52)
Clock drawing task	6.95 (0.32)
Word fluency task	17.08 (4.52)
Similarities task	10.5 (3.29)
Finger movement task	25.13 (5.84)

Table S2. Mean sub-scores of Five Cognitive Functions test (Five-Cog) in high-cognitive function and low-cognitive function groups [Standard deviation (SD)]. For clarity, values with $p < 0.05$ are shown in bold.

Sub-score	High-cognitive function group	Low-cognitive function group	t-value (p-value)
Letter-position matching task	**28.53 (4.01)**	**21.84 (4.44)**	**4.87 (<0.01)**
Category cued recall task	**17.74 (3.86)**	**14.79 (2.44)**	**2.82 (<0.01)**
Clock drawing task	7(0)	6.89 (0.46)	1 (0.33)
Word fluency task	**19.47 (4.79)**	**14.68 (2.63)**	**3.82 (<0.01)**
Similarities task	**12 (2.52)**	**9 (3.33)**	**3.82 (<0.01)**
Finger movement task	**28.53 (5.23)**	**21.74 (4.28)**	**4.38 (<0.01)**

2 NODE DEGREE ANALYSIS BASED ON PHASE LAG INDEX

2.1 Node degree based on phase lag index

The hub in a network is defined not only by betweenness centrality (BC) but also by node degree (ND), which is defined as the average of the functional connectivity of neighborhoods around the focal node in the frontal regions in the theta (4–8 Hz) band. In this frequency band, a significant strong relationship between the hub structure captured by BC in the frontal region and the Five-Cog total score was observed. In this Supplementary Material, we used ND to evaluate the hub structure in the theta and alpha bands in the frontal region.

The phase-lag index (PLI) evaluates the functional connectivity between two time series (Stam et al., 2007). The PLI is based on the asymmetry of the phase-difference distribution between the two time series, which are determined using the Hilbert transform. Designed to ignore zero and π phase differences, the PLI can reduce the influence of volume conductions. The PLI can be obtained from a time series of phase differences $\Delta\varphi$ with t_k, as follows:

$$PLI = |<\mathrm{sign}(\Delta\varphi(t_k))>|, \tag{S1}$$

where "sign" represents a signum function, $<>$ indicates the mean values, and $||$ denotes the absolute values. t_k represents a time series ($k = 1,\ 2\ \ldots,\ N$). PLI values ranged from 0 to 1. A value of 0 means no coupling or coupling with zero lag, while a value of 1 means perfect phase coupling.

In addition to PLI, we calculated the ND values. ND is defined as the average of the PLI values for each electrode node as follows:

$$ND_i = \frac{1}{n-1} \sum_{j \in N, i \neq j}^{n} PLI_{ij}, \tag{S2}$$

where PLI_{ij} is the value of PLI between nodes i and j. N is the set of all nodes in the network, and n is the number of nodes ($n = 19$).

2.2 Correlation between cognitive functions and node degree of the frontal region at the theta and alpha bands

Table S3 presents the partial correlation coefficients between the ND values and Five-Cog total score for theta frequency band and seven frontal electrodes (Fp1, Fp2, F3, F4, F7, F8, and Fz). Age and educational history were used as covariates. In any electrodes, the partial correlation coefficients did not meet the criteria of statistically significant ($p < 0.05$), in contrast to the case with BC.

Table S3. Partial correlation coefficient (rho) between node degree (ND) values and Five Cognitive Functions test (Five-Cog) total scores, with age and educational history as covariates, for the theta frequency band (4–8 Hz) and 7 frontal electrodes.

	Fp1	Fp2	F3	F4	F7	F8	Fz
rho	-0.13	0.17	-0.085	0.060	-0.10	-0.070	-0.056
p-value	0.44	0.31	0.62	0.73	0.55	0.68	0.74

3 GROUP COMPARISON OF PHASE LAG INDEX BETWEEN HIGH- AND LOW-COGNITIVE FUNCTION GROUPS

In this study, we analyzed topological characteristics of functional connectivity. In this "Supplemental Material", we additionally evaluated the adjacency matrix for the PLI value between high- and low-cognitive function groups. For statistical analysis, t-tests were conducted to assess the significantly large difference between the groups. To control for multiple comparisons, false discovery rate (FDR) correction was applied with a threshold of $q = 0.05$ using the Benjamini–Hochberg method to t-values. Figure S1 shows that the average PLIs for each delta, theta, alpha, and beta bands on the high- and low-cognitive function groups and the t-value between these groups at the four bands. In the results, no significant differences after adjustment for FDR with $q < 0.05$ were confirmed.

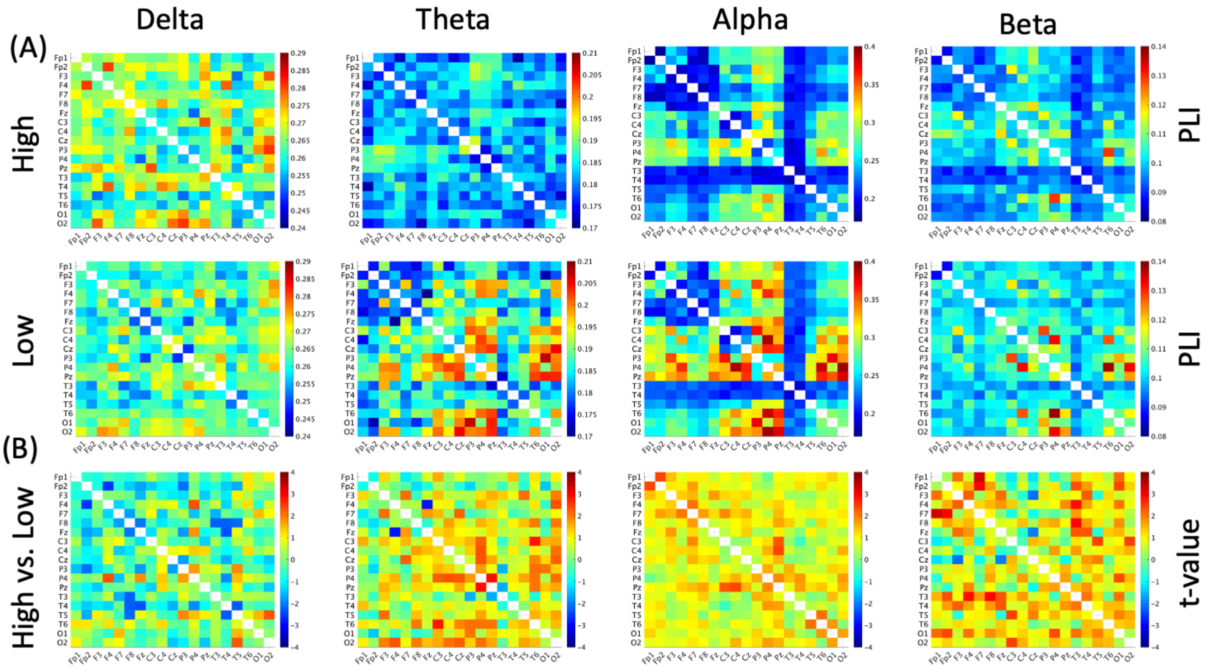

Figure S1. (A) Matrix of average phase lag index (PLIs) for each delta, theta, alpha, and beta bands on the high cognitive function group (upper) and low cognitive function group (lower). (B) The t-value matrix between these groups at 4 bands. No significant differences after adjustment for false discovery rate (FDR) with $q < 0.05$ were confirmed.

REFERENCES

Fujii, Y., Seol, J., Joho, K., Liu, J., Inoue, T., Nagata, K., et al. (2021). Associations between exercising in a group and physical and cognitive functions in community-dwelling older adults: a cross-sectional study using data from the kasama study. *Journal of Physical Therapy Science* 33, 15–21

Stam, C. J., Nolte, G., and Daffertshofer, A. (2007). Phase lag index: Assessment of functional connectivity from multi channel EEG and MEG with diminished bias from common sources. *Human Brain Mapping* 28, 1178–1193
